# Discussing the Notion of Decent Work: Senses of Working for a Group of Brazilian Workers without College Education

**DOI:** 10.3389/fpsyg.2016.00207

**Published:** 2016-02-23

**Authors:** Marcelo A. Ribeiro, Fabiano F. Silva, Paula M. Figueiredo

**Affiliations:** ^1^Department of Social and Work Psychology, Psychology Institute, University of São PauloSão Paulo, Brazil; ^2^Mackenzie UniversitySão Paulo, Brazil

**Keywords:** decent work, social constructionism, career counseling, vulnerability, Brazil

## Abstract

Despite recent stability and socioeconomic development, Brazil’s history is marked by social inequality, informality, precarious work, and psychosocial vulnerability, with little opportunity for decent and meaningful work, as recommended by the International Labour Office (ILO), for people in the country. Nevertheless, based on a social constructionist view, the hypothesis can be raised that there is no substantive definition of decent work, but rather a psychosocial one, constructed based on the discourse, narratives, and practices produced through the relational processes which grant sense and meaning to work. Therefore, the examination of narratives and discourses is an important methodological strategy to understand the socio-occupational reality of Brazil. Thus, this study aims to understand the senses attributed to working through content analysis of the narratives produced by a set of 20 urban workers and contrast them with the ILO definition of decent work, in an effort to analyze the relationships, similarities, and differences between an established collective social discourse and the interviewees’ singular narratives. The main results point out that the participants look for working with fair wages, social protection, safety, and personal development opportunities, as the ILO recommends. The main difference is that these characteristics do not derive from the State’s actions, as in the employment and formal qualification model, but come from informal sources, such as family and community relationship networks. The informal relationship networks produce job opportunities as well as social protection; qualification takes place through practical learning from more experienced colleagues; the opportunity to be able to keep working (employed or working informally) leads to success and safety; and the possibility to make choices and have control over one’s life translates into personal and occupational development. In conclusion, the participants searched for working according to the principles recommended by the ILO. Nevertheless, in contexts of vulnerability and with restricted support from the State, these principles are constructed in the community and not offered by the public power, which generates distinguished forms of decent work.

## Introduction

Touraine (2007), Castel (2009), Krein (2013), and Antunes (2015) propose that the contemporary working world has been marked by ruptures and weakening of its traditional structures and the emergence of more fluid working dynamics, making it more flexible, heterogeneous, and complex. This creates, on the one hand, room for changes and innovations but, on the other hand, frequent precariousness, instability and insecurity, as well as greater onus on the workers themselves for their working activity and career in an era of so-called contemporary stability, as stated by Ribeiro (2014).

The contemporary stability we envision refers to a form of stability generated by the current working conditions and would not be marked by instability, but by a new form of stability, achieved through the people’s active positions in their relationships with working contexts, in order to produce experiences of continuity and sense in working (Ribeiro, 2014).

In that vein, international institutions like the International Labour Office (ILO) have tried to promote safety, dignity, and protection in contemporary working, through proposals like decent work and an activity agenda to guarantee decent work: “Decent work sums up the aspirations of people in their working lives. It involves opportunities for work that is productive and delivers a fair income, security in the workplace and social protection for families, better prospects for personal development and social integration, freedom for people to express their concerns, organize and participate in the decisions that affect their lives and equality of opportunity and treatment for all women and men” (International Labour Office [ILO], 1999).

Nevertheless, the notions of security, stability, social protection, social integration, precariousness, freedom, equality, participation, decision-making power, working, and career have gradually changed, along with the senses and meanings of work, outside of variations in different contexts (Bescond et al., 2003; Auer, 2007; Mocelin, 2011; Sato, 2013; Michaelson et al., 2014; Bendassolli and Coelho-Lima, 2015; Mattos, 2015).

The concepts of meaning and sense (or meaningfulness, as proposed for this special issue), can either be considered synonyms in academic studies and research or a distinction can be made. In this article, we assume that the senses and meanings of working differ from one another, in line with the social constructionist perspective – the theoretical perspective in this research. Hence, meanings are “constructions collectively elaborated in a certain concrete historical, economic and social context. The senses, then, are a personal production deriving from the individual apprehension of the collective meanings, in the daily experiences” (Coutinho, 2009, p. 193).

Thus, sense relates to individual interpretation or the way people make meaning of themselves and their relationships with the world, for example, their working activity. This concept lies in contrast to meanings that are collective and consensual interpretations produced in a given context.

According to Bendassolli and Coelho-Lima (2015), senses are unlimited, unstable, and dynamic, while meanings are stable, category-based, reflect the accumulated cultural repertoire of a given group, and instruct its members on how to act on reality.

In this paper, we are interested in understanding the unique senses about working constructed by Brazilian workers and contrasting them with collective meanings, such as the notion of decent work of the ILO.

In line with Blustein (2006), we will use the conception of “working” instead of “work,” as “working” is a more dynamic and structuring conception that indicates action and process in a given context (verb representing an action), while “work” is a more abstract, stable and structured conception constructed in a given context and incorporated in others (substantive representing a state). Hence, “working” represents the abstract concept of “work” in action through human activities in a specific socio-historical context.

In the introduction to this article, we present several questions, inquiries, and dilemmas regarding decent work that we have derived from the literature, to be discussed based on field research in a specific socio-occupational context located in the Southern hemisphere – Brazil.

Several authors have questioned the range and universality of the notion of decent work. Rodgers (2002), Rosenfield and Pauli (2012), and Mattos (2015) defend the idea of decent work as dignified working, but question what definition of dignity the notion of decent work should rest on: universal or historically constructed as an open axiological category (Rosenfield and Pauli, 2012).

Rodgers (2002) signals the strong relationship between the concept of decent work and the notion of human dignity, as working is the sphere in which people’s economic and social objectives converge; that is, decent work is the bridge between the economic and the social (Druck, 2011). Castel (2009) stresses that working is the fundamental means to guarantee social protection and security to the majority of people without property who do not gain security from accumulated wealth.

Ghai (2002), Standing (2002), Mocelin (2011) and Mattos (2015) raise doubts on the ILO’s position that social protection and stability derive from paid working and that typical (industrial) jobs should be the model of decent work. Mocelin (2011) concludes that an *a priori* normalization of a typical job as decent work is important, but idealized and limited; after all, why would a typical (industrial) job be good if the model has been questioned for a long time? Typical jobs may lack quality and, in addition, Mocelin (2011) argues, the stability they provide does not guarantee meaningfulness and security in working, as it serves as an ambiguous indicator, because a continuous and stable job potentially represents both security and meaning, as well as stagnation and lack of sense in working.

Another important point of analysis is the contrast between decent work and precarious work. Rodgers (1989) and Evans and Gibb (2009), in a synthesis of the international literature, consider that precarious work is associated with instability or uncertainty regarding the continuity of working, lack of protection in situations of need, very bad working conditions or unacceptable occupational practices, and insufficient income, which entails social vulnerability. Barbier (2004), Mocelin (2011), Baltar (2013), and Burchell et al. (2014) criticize the dichotomy between decent work and precarious work, which the ILO generally advances from, as the contemporary complexity of the working world does not permit dichotomizations and pure types (Costa, 2010), as well as the fact that precarious work means different things in different national and disciplinary contexts (Barbier, 2004). Saunders (2003), in turn, calls attention to the fact that the international literature has used vulnerable worker as a synonym for workers in precarious situations, but that concept also leaves room for countless interpretations, due to the existence of distinct forms of occupational vulnerability (Proni, 2013).

At that, Mattos (2015) summarizes the thinking of many contemporary authors and raises the question: is there one decent work or are there distinct versions of decent work, varying in function across distinct contexts? Are there one or several ways to reach the same goal?

Ghai (2002, p. 2) states, “the decent work paradigm provokes questions about its universality and particularity.” Bescond et al. (2003) proposed that decent work means different things to distinct groups of people. Di Ruggiero et al. (2015) and Hauf (2015) indicate that there are competing discourses on decent work in different economies and contexts, because “asymmetries in power relations shape different conceptualizations of decent work” (Di Ruggiero et al., 2015, pp. 120–121).

The concept of decent work was elaborated based on consensus among groups representing different regions around the world. Its attempts to rescue full employment and renews typical work (paid work) as a model of attachment to working still exert strong influence, which excludes most underdeveloped regions that have not fully established social welfare States.

In contexts like Brazil (*locus* of study), which Ghai (2002) ranks among the developing countries with a “development model” of decent work, as opposed to industrialized countries with a “classical model” and countries moving from communism to a market economy with a “transition model,” it is important to take into account the particularities of this development. According to Costa (2010), as opposed to what happened in many of the social welfare nations, full employment has never been part of the Brazilian reality, in which a significant part of the population has always worked beyond the formal employment bonds, being active in unprotected and unregulated jobs. Therefore, one might say that informality is a matter of structural and constitutional order of the Brazilian working world, not a transitory dysfunction of that world. Recent data by the Institute of Applied Economic Research (Instituto de Pesquisa Econômica Aplicada [IPEA], 2015) show that, in Brazil, 32.5% of the economically active population (EAP) works in the informal economy (Proni and Rocha, 2010; Proni, 2013; Ribeiro, 2014).

The concept of informality or informal economies emerged in the United Nations System based on European experts’ observations of the repercussions of capitalism in African countries, as well as on studies focused on the effects of industrialization in peripheral economies, mainly in Latin America. It is understood in opposition to the idea of formality, which occurs in developed economies that are centered on employment or paid working (Cacciamali, 2000; Trebilcock, 2005; Sato, 2013). Like decent work, however, it is a controversial and polysemic concept with distinct meanings in different contexts (Cacciamali, 2000; Sato, 2013; Bendassolli and Coelho-Lima, 2015). Informality tends to be interpreted dichotomously (formal versus informal), as the negative side of paid working, a model that has also been criticized in the literature, mainly in Latin America (Noronha, 2003; Nouroudine, 2011; Sato, 2013).

Feijó et al. (2009), Nouroudine (2011), and Sato (2013) propose focusing on the positive aspects of these informal working modalities, which can offer elements to set up the social protection that is missing. The core problem of informal working is not the lack of formalization, as informal working has organization modes, agreements and rules, but the lack of social protection.

Alves and Tavares (2006), Rosenfield (2011), Proni (2013), and Tavares (2015) point out the two sides of informal working, marked by autonomy or precariousness, and often mixed up with the individualist discourse of entrepreneurism (Appay, 2005; Rosenfield, 2011) that produces undue tolerance for several forms of precariousness (Feijó et al., 2009). Castel (2009) argues that a smaller part of the population would be able to exercise modern individualism in the construction of their lives, that is, to be independent and take an individualist stand due to too many subjective investments and self-assertion (entrepreneur notion), while most of the population practices negative individualism due to disaggregation and lack of collective references of support with a consequent lack of social protection (characteristic of informality).

Therefore, according to Trebilcock (2005), in contexts like the Brazilian reality, the promotion of decent work depends on the elimination of negative aspects of informality, mainly the lack of social protection. Hence, International Labour Office [ILO] (2002, 2004), Feijó et al. (2009), Spink (2009), and Mocelin (2011) acknowledge that there exists no clear border between formal and informal working, but a *continuum* ranging from extreme lack of protection and precariousness to extreme protection with intermediary levels, producing degrees of formality and informality. “Our working hypothesis is to assume that, just like the border between formal and informal working is not well drawn, in the informal sector, one can also identify a *continuum* of situations in which the premises of decent work are more or less present” (Feijó et al., 2009, p. 331).

According to Druck (2011), in the Brazilian reality, several types of precariousness exist, such as vulnerability associated with social participation and inequality, intensification of working and outsourcing, insecurity and occupational health, loss of individual and collective identities, weakening of workers’ organizations and disposal of Labor Law. “Unemployment, low wages, informality and lack of protection are severe problems that affect a significant part of the Brazilian workers” (Proni, 2013, p. 826), despite distinct degrees of vulnerability and occupational precariousness in Brazil.

That is the picture in which, in Brazil, the study of informality has gained room, according to some recent studies (Spink, 2009; Abramo, 2010; Costa, 2010; Araújo, 2012; Araújo and Lombardi, 2013; Dedecca and Menezes, 2012; Sato, 2013; Antunes, 2015; Bendassolli and Coelho-Lima, 2015; Tavares, 2015). These studies privilege specific angles on the phenomenon, such as social relationships (sociability), incomes, working conditions, gender and race relations, subjective wellbeing, health, quality of life, meaningfulness in work, organizational processes and, according to Bendassolli and Coelho-Lima (2015), predominantly the consideration of the dynamism of informality and its extensive multiplication – including informal practices as a part of the formal economy (Costa, 2010; Krein and Proni, 2010; Tavares, 2010; Antunes, 2015), or the concept of new informality (Noronha, 2003).

In that sense, in contrast to many studies the ILO has published, the soundest route would be the creation of decent work in the informal economy, rather than the elimination of informality in order to achieve decent work, as Tokman (2009) highlighted. After all, poorer or developing countries often have a heterogeneous and important informal economy that should be maintained. “However, it should not be a job at any price or under any circumstances” (Trebilcock, 2005, p. 3), but the four dimensions of decent work should be minimally guaranteed (rights, employment, social protection, organization and social dialog). Hence, according to Ghai (2002), the paradigm of decent work is universal, but requires contextualization for the sake of effectiveness, as each country needs to find its own way to produce decent work (Boyer, 2006), a notion that highlights the importance of social dialog for the conception of decent work (Rodgers, 2002).

“This is expressed today as decent work for all, whether the activity is carried out in a formal or an informal context” (Trebilcock, 2005, p. 1). Thus, the strategies to guarantee decent work and fundamental rights to informal workers should aim to accomplish the same objectives (Hanssene, 1999), which, according to Chen et al. (2004), are promoting opportunities, securing rights, promoting protection, and promoting voice. “To the previously consolidated notion of a high-quality job, the concept of decent work adds the notions of rights” (Abramo, 2010, p. 152).

In summary, as discussed to date, the main issues associated with studying decent work in Latin America, and specifically in Brazil are: (a) What notion of dignity is associated with decent work? (b) Should decent work depart from the typical employment model? (c) Should a generic or a contextualized conception of decent work be adopted, with the consequent need to relativize the concepts for the sake of a psychosocial discussion of decent work? (d) Does decent work exist in the singular (universal concept) or plural (singularized concepts)? (e) Should decent work be created in the informal economy or should informality be eliminated for the purpose of decent work?

In view of these inquiries and to understand the matter of decent work in the Brazilian reality, we consider the contributions of Richardson (1993), Ghai (2003), Blustein (2006, 2011), Deranty and MacMillan (2012), Michaelson et al. (2014), and Bendassolli and Coelho-Lima (2015), and argue in this paper for the importance of including the psychosocial dimension in an assessment of whether a work is decent or not and defending “a more nuanced analysis of the activity of work based upon the subjective investment that people make in their work to determine whether the work experience is a decent one or not” (Di Ruggiero et al., 2015, p. 126), which lead us to the analysis of the relational co-construction of the senses and meanings of working in a given context (Blustein, 2011; Duarte, 2015; Savickas, 2015).

Michaelson et al. (2014, p. 77) affirm that “in the human quest for meaning, work occupies a central position.” Together with Tolfo and Piccinini (2007) and Bendassolli and Gondim (2014), they point out that, traditionally, the senses and meanings of working were constituted as themes different authors have investigated from the perspective of several epistemological branches and based on different psychological and psychosocial phenomena produced by the quality of life at work (Hackman and Oldhan, 1975). This phenomenon has been studied in different ways. First, researchers have looked at the results of working and desirable organizational consequences, such as job satisfaction, engagement, well-being, work values, work involvement, work orientation, job performance, organizational citizenship behavior, organizational commitment, and occupational identification (Michaelson et al., 2014). Second, studies have focused on the articulation of variables like importance, regulation, and resulting from working (Meaning of Work International Research Team [MOW], 1987), or significance, orientation and coherence (Morin, 2001). As pointed out earlier in the text, here we use the concept of sense to define what working simply means to each person.

In this research, the focus is to understand the senses attributed to working so as to be able to compare these senses with the meanings produced by institutional discourses, such as the ILO’s notion of decent work (International Labour Office [ILO], 1999). This procedure aims to analyze the correspondences and incongruences between the narratives of workers from a specific context and generic discourses of international institutions since, in line with Kaplan (2002, p. 180), “Policy instruments operate as rhetorical and normative modes of discourse to convince others to take action.”

Thus, we will attempt to understand if a given working activity is “meaningful, that is, is purposeful and significant” (Michaelson et al., 2014, p. 79), since “the fact that work has a particular meaning does not necessarily determine that it is meaningful… meaningfulness refers to the amount of significance something holds for an individual” (Rosso et al., 2010, p. 95). Therefore, in this article, we will take “sense of work” as a synonym for “meaningfulness in working.”

Burchell et al. (2014) and Sehnbruch et al. (2015) emphasize that the term “decent work” has not been used in the academic literature and that psychology has focused more on studying working as employment, often without including multiple coexisting forms of working. Thus incorporating the concept of decent work into the psychological literature constitutes a great opportunity to investigate, assess, and understand phenomenon like informality or the informal economy, which are characteristic of the Brazilian reality (Amin and Singh, 2002; Sato, 2013).

Bendassolli and Coelho-Lima (2015) indicate that exploring the meanings of informality can be one way to discover the symbolic/cultural meanings produced by/in capitalism with regard to informality. These explorations can reveal positive meanings institutionally produced about informality (entrepreneurism, self-initiative, autonomous, and eventually informal forms of working), as well as depreciative meanings in the sense of informality being defined as the negative of formal employment (Sato, 2013) through the macro-narrative of formal employment.

Hence, we can hypothesize, from a social constructionist view, the theoretical perspective of this study, that decent work should not be substantively but rather psychosocially defined. This definition must be constructed based on the discourses, narratives, and practices produced by the workers themselves through the relational processes of signification that grant them senses (or meaningfulness) and meanings. This method of definition allows for distinct versions of decent work to vary as a function of distinct contexts.

Blustein (2011, p. 4) proposes that the notion of sense in working “includes one’s sense of purpose in working and the way in which one understands his/her work life.” Richardson (1993, p. 427) complements this view by proposing that the “focus on the study of work in people’s lives in which work is considered to be a central human activity that is not tied to or solely located in the occupational structure” and Flum (2015, p. 148) points out that “to work is to relate … A sense of being-in-the-world is based on interaction. It is a combination of an awareness of connection and meaningful action.” The authors agree that sense “is given shape in social interactions in which one’s own constructions of working are embedded in relational understandings” (Blustein, 2011, p. 4) as part of a process of self-construction (Guichard, 2009) or life construction (Duarte, 2009). Savickas (2005, p. 44) states that: “rather than choose among attractive options, some individuals may have to take the only job that is available to them, often a job that grinds on the human spirit because its tasks are difficult, tedious, and exhausting. Nevertheless, the work that they do can be meaningful to them and matter to their community.”

Therefore, as also suggested by Spink (2007), we should concentrate on understanding the daily life of “unseen” people, asking about the theories and practices that guide their lives, as well as their conceptions of dignity and citizenship, in an attempt to answer what decent work means to them. We must not be constrained by an idealized view of paid and stable working, but focus on what we have – whether we like it or not – because it is extremely important to “give voice to those who have not much voice in our research” (Blustein, 2006, p. 307). As pointed by Savickas et al. (2009, p. 243): “If there exist multiple ways to interpret one’s own diverse life experiences, different life perspectives and designs become possible.”

To accomplish this task, this study is based on three basic conceptions proposed by social constructionism. First, the psychosocial element is central and can be defined, based on Frosh (2012), “as a process that is neither ‘psychological’ nor ‘social,’ but transcends the separation of these elements to create something new – the psychosocial” (Ribeiro, 2015, p. 20). Second, social constructionist thought is based on the notion of relational ontology according to Gergen (1997), who states that the “reality is not objectively constituted, but intersubjectively constructed by means of the narratives and social practices generated in relational processes” (Ribeiro, 2015, p. 20). And, finally, it is based on the notion of narratability proposed by the life designing paradigm (Savickas et al., 2009) and understood as the psychosocial capacity to meaningfully narrate one’s life history to oneself and others.

According to Brockmeier and Harré (2003), narratives constitute an important methodological strategy for understanding the working world through the people who act in it. These narratives potentially define the ways in which people construct senses in their psychosocial relationships, turning them into narrative realities in line with Savickas et al. (2009), which would allow analysis of the multiple forms and meanings of the current social discourse.

The main objective in this study was to understand the sense of working for a group of 20 formal and informal workers in the city of São Paulo, Brazil and contrast them with the ILO’s definition of decent work, aiming to analyze the relationships, similarities, and differences between an established collective social discourse and the interviewees’ singular narratives, as well as to contribute to the discussions on the notion of decent work and its possibilities and limits in terms of theoretical conceptions and practical applications. Thus, we intend to discuss the following research problem: How do workers from socio-occupational contexts marked by inequality, informality, instability, and precariousness construct senses in their working experience?

## Materials and Methods

In this research, the qualitative narrative approach (Polkinghorne, 1988) is used because of the minimal existing literature on the subject and the need to explore the fieldwork to formulate new hypotheses from the everyday experience and senses related to working built by people throughout their lives. The main reason for using narrative interviews is that they can capture the lived experience of working both in terms of the construction of working life over time, as well as the senses constructed in relation to work. In this regard, the models of quantitative research and qualitative research based on pre-existing categories of analysis would not be appropriate to achieve the objectives of this research.

The qualitative narrative approach (Polkinghorne, 1988) was used to co-construct the life histories and significant experiences in the participants’ daily working, stimulating their unrestricted speech and allowing them to direct the narrative. It aimed to exploring the basic points of the ILO’s definition of decent work (International Labour Office [ILO], 1999), which are: opportunity for work with fair income, security in the workplace and social protection, prospects for personal development and social integration, and respect for fundamental rights. The singular autobiographical narratives of life and daily working the participants co-constructed with the researchers permitted contact with the way each person was influenced by social relationships and discourses. They reconstructed the senses and strategies they used as a function of the properties of the narratability, that is, the ability to narrative one’s life history with a sense of identity and psychosocial legitimacy, in line with Savickas et al. (2009).

Alvesson and Willmott (2002) and Bendassolli and Coelho-Lima (2015) highlight that, when studying singular narratives, one necessarily gets in touch with the way each person intersects with social relationships and discourses, creating a space for the emergence of social discursive productions; the autobiographical narrative is a singularity in which the others can see themselves through social discursive productions, which construct the collectively shared meanings. The reliability of a personal narrative would therefore derive from the fact that it is constituted based on collective agreements constructed inside a given community that shares central social discourses, which are produced with and related to common meanings and practices (Denzin, 1989).

Hence, the study did not seek statistical generalizations and is more based on the validity of the analysis than on the representativeness of events and the confirmation among methodologically similar studies undertaken previously. “The advantage of using qualitative methods is that we have an opportunity to learn about new concepts and experiences in relation to working that are above and beyond what we already know” (Blustein, 2006, p. 232).

### Socio-Occupational Context of the Participants

First, the participants’ socio-occupational context needs to be characterized – the city of São Paulo, which is the largest Brazilian city with approximately 12 million inhabitants, is located in the Southeast of Brazil, which, together with the South, is responsible for 71.8% of the Brazilian Gross Domestic Product (GDP). It is considered a globalized, multicultural city with large groups of immigrants (both from other countries and other Brazilian regions). It is also a wealthy city, and is the main financial and corporate center of South America, based on industries and services, with plenty of working opportunities. Moreover, it is marked by a more collective than individual relational tradition (Brewer and Chen, 2007), in which social embeddedness is very meaningful to people’s experiences.

In educational terms, the achievement rates in São Paulo are comprised as such: 33.4% have a primary school education, 19.7% a middle school education, 29.3% are high school graduates, and 17.6% have graduated from college (International Labour Office [ILO], 2013).

In terms of the job market, 7.4% of the population is unemployed, 64.9% are engaged in the formal job market, and 27.7% are informal workers, with a mean monthly income of US$365. Ninety percent of new jobs in the formal market demand at least a high school education. In recent decades, there has been a gradual increase in people working with greater social protection due to the socio-economic development of the country. However, the research participants, due to the length of their careers, have had little benefit from this national improvement.

The context is also marked by great socioeconomic inequality (sixth city in the world in terms of number of billionaires but with 10% of the population living below the poverty line), spatial segregation and contact between formal and informal economies. This inequality creates an important space in which we can analyze the role of decent work in contexts not predominantly marked by the typical employment model.

### Participants

The participants were recruited using intentional selection at a college career counseling service and each of them met the inclusion criteria of being: urban workers, without a college education, from São Paulo, Brazil, with at least 20 years of (formal and/or informal) working experience, who were working at the time of the research. The criterion of having at least 20 years of working experiences was included to understand the changes and continuities in participants’ lives and daily working histories, in terms of the personal senses constructed and the prevailing social meanings, which would not be possible in shorter periods of working experience.

The number of participants was not defined *a priori*, but during the data collection phase, based on the rules of representativeness (differently from the sample, the goal is to highlight significant informants on the investigated theme), homogeneity (minimal common criteria to choose the participants) and saturation, when repeated narratives started to be found (Bardin, 1977).

The choice of this specific set of participants was not with the intention of being representative of Brazilian low-skilled workers, but aimed to highlight significant informants on the investigated theme, mainly because they were a group of understudied workers who sought a career counseling service, and were clearly eager to discuss the working world. In addition, the main difference between workers who qualified for the study and those who did not is that, in general, understudied workers hold informal jobs and skilled workers hold formal jobs. Thus, the sample was driven by the interest of the current research in working experiences not guided by the logic of typical work as a model for decent work.

The set of participants is formed by 20 urban workers (10 men and 10 women), without a college education, between 35 and 55 years of age, from São Paulo, Brazil, with 20–40 years of working experience. Most participants were married, mulatto^1^, between 41 and 50 years of age, had completed middle school, had between 31 and 40 years of working experience beginning at the age of 10–15 years, varied between formal and informal working activities in the course of their career, and were working at the time of the research, mainly as blue-collar workers, domestic servants, doormen, and street hawkers, as it can be seen in **Table 1**. It is important to note the division of working by gender; women were more likely to be domestic servants and street hawkers, activities characterized as more flexible, while the men were more likely to be blue-collar workers and doormen, activities that traditionally have more fixed work hours, supporting a trend in the Brazilian job market toward these occupations (Instituto de Pesquisa Econômica Aplicada [IPEA], 2015). Their main motivation to participate in this study was to contribute to the improvement of the career counseling service they were attending to rethink their careers.

**Table 1:** Characterization of the set of participants.

Features	Participants (*n* = 20)
Gender	Men – 10 (50%)
	Women – 10 (50%)
Skin color	Black – 5 (25%)
	Mulatto – 11 (55%)
	White – 4 (20%)
Marital status	Married – 17 (85%) Single – 1 (5%) Divorced – 1 (5%)
	Widow – 1 (5%)
Age groups	30–40 years 5 – (25%)
	41–50 years 10 – (50%)
	≥51 years 5 – (25%)
Educational level	Elementary school – 5 (25%)
	Middle school – 5 (25%)
	High school – 10 (50%)
Career length	20–30 years – 9 (45%)
	31–40 years – 11 (55%)
Career beginning	5–10 years old – 4 (20%)
	10–15 years old – 10 (50%)
	≥16 years 6 – 6 (30%)
Predominant kind of working model over the career	Formal economy (Private sector employee) – 6 (30%)
	Informal economy – 4 (20%)
	Mix of working models (formal/informal) – 10 (50%)
Current working activity	Blue collar worker – 5 (25%)
	Housekeeper – 5 (25%)
	Doorman – 4 (20%)
	Street vendor – 2 (10%) Others – 4 (20%)

### Instruments and Procedures

The instrument used was the thematic autobiographical narrative, defined as an open-ended report on the participant’s working life history, which the researchers can consult for clarifications, complementation, further development, and approach of unexplored themes (Van Langenhove and Harré, 1993). After the institutional authorization to pursue the research and with the participants’ consent, the interviews were held at the college career counseling service the participants were attending. Each interview took between 60 and 180 min. The interviews started with the following request to the participants: “Please, tell me about your daily working experiences in the course of your history.” First, the participants talked about their daily working life experiences through free speech, in which the people recounted their own history (autobiography) and, in the course of the narratives, the researchers intervened as necessary for explanations, additional information, and unexplored issues, creating a co-construction process of the autobiographical narratives (heterobiography). The complete interviews were then submitted to content analysis of the autobiographical narratives.

The interviews were recorded as an audio file, the dialog was transcribed, and then analyzed as described below.

### Analysis of the Narratives

Based on descriptive narrative investigation (Polkinghorne, 1988), the narratives were submitted to content analysis as proposed by Bardin (1977), in combination with additional suggestions by Van Langenhove and Harré (1993), through the following steps:

#### (a) Pre-analysis Phase

Individual independent reading of each transcription of the autobiographical narratives to organize a content structure for the personal senses and collective meanings that informed on the participants’ life histories and the significant daily working experiences, making them operational in terms of the exploration of the earlier mentioned basic points in the ILO’s definition of decent work (International Labour Office [ILO], 1999).

#### (b) Vertical Analysis

Identification of each participant’s key indicators, as well as a survey of registration units that represented the key indicators. The registration units are the smallest content parts expressed by phrases or ideas extracted from the narratives, and an indicator is understood as content that can summarize the main elements of a given psychosocial phenomenon, like is the case of the senses of working in this study.

#### (c) Horizontal Analysis

Grouping of the narratives based on their shared content (registration units and indicators), resulting in the identification and systemization of the fundamental social discourses of the specific context studied on decent work and their respective indicators. These were contrasted with the ILO’s definition of decent work (International Labour Office [ILO], 1999), with the aim of analyzing the relations, similarities and differences between a universally established collective social discourse (ILO), the fundamental social discourses of a given specific context (meanings of decent work in Brazil) and the interviewees’ singular narratives in this specific context (senses of decent work for a set of Brazilian workers).

An intercoder reliability system was used in which three independent judges identified and categorized the contents of the participants’ narratives according to the research objectives, seeking the intercoder agreement between them (Tinsley and Weiss, 1975) that resulted in the emic categories listed in the results section.

### Ethical Standards

This study was carried out in accordance with the recommendations of the Institutional Ethics Committee with written informed consent from all participants. All participants gave written informed consent in accordance with the Declaration of Helsinki and with Resolution 466/12 by the Brazilian Committee for Research Ethics (Conselho Nacional de Saúde [CONEP], 2012).

## Results

The results are presented and discussed based on the shared senses of working the participants constructed within the specific context of Brazil (senses of decent work for a set of Brazilian workers based on their singular narratives crossed and co-constructed by the fundamental social discourses of the meanings of decent work in Brazil). These senses will gradually be contrasted with the central principles of the ILO’s conception of decent work (International Labour Office [ILO], 1999), which is the universally established collective social discourse on decent work and is comprised of: (a) Opportunities to work with fair income; (b) Security at work and social protection; (c) Perspectives of personal development and social integration; (d) Freedom of expression, organization and decision; and (e) Respect for fundamental rights. The author of the vignette will be characterized between parentheses in each excerpt of the singular narratives cited (for example, P1, man, 45 years – participant 1).

We chose emic categories, that is, categories constructed based on excerpts of the singular narratives, to summarize and name the main senses of working. These are: (a) Without at least having the possibility to choose; (b) Everything I have learned, I did on my own; (c) I met a person who invited me to work; (d) I accept what is coming, because my income has never been very good and success means to keep on working; (e) It seems that everything is always temporary, remuneration is always unsatisfactory and the working is hard; (f) It’s working and making money, I don’t think about the future; and (g) Decent work is …

### Without at Least Having the Possibility to Choose

The large majority of the interviewees said that they started their working trajectory in childhood or youth in the North and Northeast of Brazil as a form of financial help to the family, generally in a small family business and often some type of informal work. As the opportunities in their natal city were restricted, migration to the Southeast of Brazil provided an option to be able to work, both for participants who grew up in urban areas (“I migrated from the small city to the big city to be able to work” – P1, man, 45 years), as well as in rural zones (“I migrated from the farm to the big city to be able to work” – P2, man, 35 years).

The beginning of participants’ professional working trajectories were marked by an inability to choose and decide on what work to engage in, as described by P4 (man, 36 years), who said that he started “to work due to family pressure and, since then, [is] choosing what comes up” and P5 (woman, 49 years), who said that: “I did not choose this profession, it was the job that came up.”

This working trajectory start seems to determine the sequence of the subsequent trajectory of the majority, as represented in some statements. P3 and P2 talked about never stop working. P3 (man, 50 years) said: “I started early and I have never stopped working,” as well as P2 (man, 35 years) told: “without at least having the possibility to choose … you can never stop working.” P3, P12, and P16 talked about the impossibility to choose. P3 (man, 50 years) stated: “In fact, I did not choose a profession, but the circumstances led me to this option,” P12 (man, 50 years) said: “I did not choose any of the jobs I had, I always had to accept the opportunities that emerged on each occasion,” and P16 (man, 52 years) declared: “I accepted anything that would come up, that came up.”

In summary: “I have always worked on what came up without being able to choose anything” (P3, man, 50 years), began “a story of many difficulties and instabilities, I don’t know where I made a mistake or what would be the right course” (P8, man, 56 years). Although “work is good, I have nothing to complain about, I will have to work until the end of my life” (P10, woman, 38 years).

Castel (2009) considers that the possibility of making choices is directly related to power in social relationships and that, for a large part of the population who does not have choices, what remains is negative individualism, marked by a lack of social support, in which choices are determined by psychosocial contingencies, not people’s decisions. The lack of opportunity to make choices and decisions marked by “doing what comes up” (P16, man, 52 years) produces polyvalences due to negative individualism. On the one hand, this situation provides a flexibility that guarantees employability and, on the other hand, it often makes the sense of working and of making any kind of planning impossible, turning the future into a reproduction *ad infinitum* of the present, as discussed by P2 (man, 35 years): “My future perspectives are to continue in the same thing I am doing now.”

This long-term circumstance conflicts with two principles of decent work as recommended by the International Labour Office [ILO] (1999): perspectives of personal development and social integration and freedom to decide. Further, this state truly seems to worsen the working experiences of the interviewees, in accordance with Rodgers (1989) and Evans and Gibb (2009).

### Everything I have Learned I did on My Own

If the trajectory of working starts early and if it is marked by difficulty choosing and defining what work to engage in, there is also an alternative form of learning embedded in this trajectory. This learning is not centered on education in schools, but is practical learning, which most participants value. For example, they say “I haven’t studied and I don’t miss that” (P1, man, 45 years) and “I don’t see that much need for a college degree in the work I do” (P4, man, 36 years), breaking with the dominant logic that education would prepare individuals for working and that good performance is directly related with previous specialized learning. “I never imagined that I would work in what I have worked and I think I did not even know what that was when I went to work the first day, but I have always received compliments for my performance at working” (P5, woman, 49 years).

The majority of participants says that “everything I have learned I did on my own”; “my main source of learning was the errors, trials and errors … on my trajectory there were many things I found hard to achieve and which I was able to do” (P8, man, 56 years). P1 agrees, stating: “I introduced myself to the owner of the business, I said I was able to do anything. I got the job, but I didn’t know exactly what I was gonna do … I have always learned everything in practice and I’ve always been successful” (P1, man, 45 years).

Most participants share this feeling of being able to learn in practice. P10 summarizes this shared perception well: “I feel proud of myself. Sometimes I think, can I handle it? Did I handle it? Did I manage? So I feel proud of myself. I didn’t study but I managed, like many others I know who have done the same … And I feel proud of myself, yes” (P10, woman, 38 years). Again, according to Castel (2009), it is negative individualism, due to a lack of social and State support; not individualism due to too many subjective investments and assertions of self (notion of entrepreneur), but an alleged autonomy established through threats to opportunities for working and studying, as described by Alves and Tavares (2006); Rosenfield (2011), Proni (2013), and Tavares (2015).

The International Labour Office [ILO] (1999) discusses personal development perspectives but based on the logic of institutional formality, and the State’s responsibility to provide learning conditions. In the cases reported, however, learning is a construction of the person himself/herself in relation to his/her context. Therefore, it is a psychosocial construct (Ribeiro, 2014), which depends on the informal relationship network: “I feel happy for overcoming the difficulties alone, with the help of my family only” (P9, man, 51 years).

### I Met a Person Who Invited Me to Work

There is a significant point in the senses the participants’ working life that is distinct from what the ILO’s conception of decent work proposes; the workers need a support network for their trajectories and working life, but it should be provided formally by the State, instead of informal relationship networks. In countries like Brazil, where the occupational care network is not structured and comprehensive enough to reach everyone, informal networks help to solve the issues of working life. Further, they go beyond the realm of working, constituting true relationships of solidarity in light of the partial absence of the State.

P1 (man, 45 years) says that, “whenever I leave a job with something at hand, even when that did not happen, I was able to get a job rapidly because of my friends,” emphasizing that everything he achieves in his personal and working life comes through indication and friendship – “everything is an exchange of favors.” The base for the protection and security to keep working is his ability to use his contacts, in accordance with the idea of Flum (2015, p. 148) that “to work is to relate.”

P4 (man, 36 years), like other participants, said that he did not turn to the public occupational care network in situations of unemployment, but instead relied on the informal contact network: “after a while, a friend introduced me to his friend who invited me to work” (P8, man, 56 years). Therefore, to keep working, “you need to have a lot of friendships, if you’re a good person to others, you achieve many things” (P13, man, 50 years). Therefore, many people maintain and participate in support networks that work “to get formal as well as informal working activities” (P6, woman, 38 years), as “the type of work does not matter, what matters is to keep on working” (P10, woman, 38 years).

### I Accept What is Coming, Because My Income has Never been Very Good and Success Means to Keep on Working

Most participants switched between formality and informality in the course of their working trajectories, without any preference for one or the other working modality, with the mere need to “keep on working” (P9, man, 51 years). To continue working is what guarantees security and stability, in line with Castel (2009)’s proposition that working is fundamental to guaranteeing social protection. Success and security are perceived as the ability to continue working, whether formally or informally, distinctions that do not seem to be as clearly divided as the literature proposes. Indeed, the participants’ experiences support the idea of a *continuum* from extreme lack of protection and precariousness to extreme protection with intermediary levels, creating degrees of formality and informality, as stated by International Labour Office [ILO] (2002, 2004), Feijó et al. (2009), Spink (2009), and Mocelin (2011).

The participants gave several examples of vacillation between formal and informal working activities along their trajectories. P1 points out (man, 45 years): “One day they needed a person for a 3-day job in a bar and I accepted. I ended up reconciling the two jobs, one formally registered and the other not,” as well as P6 (woman, 38 years): “when I am not formally employed in companies, I do the weekly cleaning at houses in the city where I live.” P2 (man, 35 years) stated that: “This thing with formal and informal, I’ve never understood it very well, because there are good and bad things to any job,” as well as P1 (man, 45 years) who said that: “We had to win in life … success is the ability to pay all expenses without getting unemployed,” and P4 (man, 36 years) who explained that: “without a job, then there’s a problem, but when I don’t have a job, I turn to acquaintances and they help rapidly.” These quotes align the informal support network for working with the idea argued by Feijó et al. (2009), Nouroudine (2011), and Sato (2013) that the main problem with informality is the lack of social protection it affords and that the promotion of decent work depends on the elimination of the negative aspects of informality (Trebilcock, 2005).

Hence, the idea that social protection and stability exclusively accompany paid working and that typical (industrial) employment should be the model of decent work does not seem to resound with the participants, supporting the criticism by Ghai (2002), Standing (2002), Mocelin (2011), and Mattos (2015). In that sense, in response to one of the questions raised in the Introduction about the relationship between decent work and informality, it seems the participants indicate the need to create decent work in the informal economy, instead of the need to eliminate informality for the sake of decent work. This argument validates the idea that informality is a structural and constitutional matter of the Brazilian working world (Proni, 2013; Instituto de Pesquisa Econômica Aplicada [IPEA], 2015).

### It Seems that Everything is Always Temporary, Remuneration is Always Unsatisfactory, and Working is Hard

Despite this apparent lack of distinction between formal and informal working for decent work, stability always seems to be temporary, in terms of contemporary stability, in line with Ribeiro (2014) and Sultana (2013). As P2 reports (man, 35 years): “I have never experienced great moments of stability in life, it seems that everything is always temporary;” it is as if “I had no control over my career” (P5, woman, 49 years); and, although “working is good, you cannot complain, but I’ll have to work until the end of life” (P10, woman, 38 years). Stability seems to be a synonym for “keeping on working,” as P9 summarized well (man, 51 years), which is guaranteed by informal support and relationship networks.

In addition, there are great difficulties in gaining appropriate remuneration, which conflicts with the principle of opportunity to work for a fair income in the ILO’s recommendation (International Labour Office [ILO], 1999). P2 demonstrates this point (man, 35 years): “remuneration has never been totally satisfactory, right?” P7 says (woman, 45 years): “my career ambition is to increase my income, but I end up accepting what comes, because my salary is never that good,” and P13 describes (man, 50 years): “I leave one job because the salary is low and because I think I would get something I thought would be better.” In short, the main problem is the existence of “great rush and working, little financial gain” (P13, man, 50 years).

Besides the unsatisfactory remuneration, working journeys are intense and working activities are very hard: “There is little time for leisure and personal life, I work every day, almost without leave” (P1, man, 45 years), “Ah, I really find myself in a mad life, I never get time to relax” (P14, woman, 50 years). These experiences may cause withdrawal: “I spent two and a half years there and I resigned, I was working a lot” (P17, woman, 42 years) and are often, marked by a double working journey. For example, “I used to work as a guard in the condominium and, after my shift, I used to go to the trailer and sell my things” (P9, man, 51 years). These double working experiences are generally aiming to offer more comfort to the family: “After all, all the conquests I have made and what I am able to give to my daughters comes from 16 h of continuous working in this trailer selling things” (P9, man, 51 years).

Further, there is the gender issue, marked by the double shift (working and household chores) characteristic of the so-called bipolarity of women’s working (Bruschini and Lombardi, 2000). This phenomenon appeared in the reports of P14 (woman, 50 years): “And there’s something else, besides working out of the house, you have to work at home,” and of P17 (woman, 42 years): “Women still have to work out and at home.” The double shift is a chronic problem of unequal opportunities and working conditions for men and women pointed out by the International Labour Office [ILO] (1999, 2004, 2013) and discussed by Abramo (2010), Araújo (2012), and Araújo and Lombardi (2013) who highlight the considerable differences in income and hours dedicated to domestic work between men and women.

This situation conflicts with another principle recommended by the International Labour Office [ILO] (1999), the respect for fundamental rights, which, in this case, includes the right to relax from working. Further, this situation seems to represent a precarious working situation, due to the instability or uncertainty about the continuation of working, lack of protection in situations of need, and insufficient income (Rodgers, 1989; Evans and Gibb, 2009; Proni, 2013).

### It’s Working and Making Money, I Don’t Think About the Future

This situation influences individuals’ view of the future, which the majority of participants often did not conceive of as a possibility. Instead, the perceptions of the future are based on present tasks and achievements: “I am unable to think of tomorrow, to make plans, those things. I am unable to do that. I let things come, I don’t think, I don’t keep on making plans, now I’m gonna do this or that” (P10, woman, 38 years), “I think more of the present and of keeping on working” (P9, man, 51 years), after all “my future perspectives are to continue doing the same thing I am doing now” (P2, man, 35 years).

The participants’ future predictions can be summarized in two ways. On the one hand, there is the material aspect of improving one’s remuneration, as described by P1 (man, 45 years): “I intend to increase my monthly income,” and P10 (woman, 38 years): “It’s working and making money, I don’t think about the future.” On the other hand, there is the search for meaningfulness in working and control over one’s trajectory, as articulated by P1 (man, 45 years): “I want to develop a job to work alone, being more independent,” and P6 (woman, 38 years): “I would like work that paid more and offered more professional accomplishment.” In response to missing a working identity, some participants agree with P8 who said, “when analyzing my trajectory, I am unable to identify an identity, a theme to say it is mine” (P8, man, 56 years), although some conform to the situation: “I don’t think of what my future would be, I am not qualified for a better job” (P5, woman, 49 years) and “I regret not having been able to save money in the course of my life” (P1, man, 45 years).

Hence, in both formal work and informal work, the notion of dignity seems to be equally based, relationally co-constructed and not established *a priori* in terms of material conquests (fair income) and one’s accomplishments in working (senses of working), in accordance with Richardson (1993), Blustein (2011), and Flum (2015).

### Discussion: Decent Work is …

In synthesis, each participant constructed a singular view on what decent work is supposed to be, which varied across several psychosocial dimensions of working life, some in tune with the ILO’s decent work concept (International Labour Office [ILO], 1999), others not.

There are four singular views that support the ILO’s concept of decent work (International Labour Office [ILO], 1999). The first is related to the dimension of future protection. According to P1 (man, 45 years), “decent work allows you to save money in the course of your life” and P10 (woman, 38 years), “decent work means to be able to have a future after you stop working” (social protection according to the ILO). The second is the dimension of the opportunity to make decisions and have some degree of control over one’s life. As P2 stated (man, 35 years), “decent work is to be able to make choices,” and P7 agreed (woman, 45 years), “decent work is to have control and autonomy in life” (freedom to decide according to the ILO). The third is the dimension of security and good income. According to P11 (man, 47 years), “decent work is to have a fixed job and make good money,” P12 (man, 50 years), “decent work means security, even if temporary,” P13 (man, 50 years), “decent work means autonomy, security and remuneration,” and P16 (man, 52 years), “decent work means having security, right?” (security at work and opportunities to work with a fair income according to the ILO). And, finally, the fourth is the dimension of the de-intensification of working, as described by P14 (woman, 50 years) and P17 (woman, 42 years), “decent work means calmer work” (respect for fundamental rights according to the ILO).

There are three singular views that did not fully support the ILO’s concept of decent work (International Labour Office [ILO], 1999). The first is the dimension of career stability, described as being able to work continuously, as articulated by P4 (man, 36 years) and P5 (woman, 49 years): “decent work is being able to keep on working.” The second is the dimension of possibility of meaningfulness in working, mainly expressed by P6 (woman, 38 years), “Flexibility guarantees work, but withdraws the possibility of meaningfulness in working,” and P8 (man, 56 years), “decent work is being able to find an identity in one’s work.” The third is the observation by P9 (man, 51 years) that, in contexts like the Brazilian working world, “decent work is being able to overcome difficulties, even without the support of the State.”

In summary, the participants’ working trajectory starts early in childhood and youth, is marked by a sequence of non-chosen, badly paid working activities that make up an intense working journey. Learning occurs informally as part of working practice, and is developed with little valuation of formal education and great acknowledgment of personal efforts. Informal relationship networks and support constitute the psychosocial base for security and for the conquests of working life, which generally is a movement of transition between formality and informality. Hence, the factors that grant meaningfulness in working and could be described as characteristics of decent work are appropriate remuneration, being able to continue working, having an informal support network, being polyvalent, being able to make choices, and having control over one’s trajectory.

This presentation about diverse and relationally constructed senses of decent work, which intersect with one another, supports the need to relativize the concepts with a view to a psychosocial discussion of decent work, producing not a generic, but a contextualized concept of decent work. After all, according to Bescond et al. (2003), Di Ruggiero et al. (2015), and Hauf (2015), decent work has different meanings for distinct groups of people, demonstrated by the need to take into account the particularities of how working is developed in Brazil, because “asymmetries in power relations shape different conceptualizations of decent work” (Di Ruggiero et al., 2015, pp. 120–121).

Despite the need for contextualization, universal elements and dimensions are equally important as guidelines. There exists a tension between the universal and particular nature of the decent work concept; “on the one hand, the existence of an absolute and universal value, inherent in all human beings anytime and anywhere; and, on the other hand, the particular nature” (Rosenfield and Pauli, 2012, p. 322). This tension is marked by several concepts of decent work that more concretely refer to cultural and historically determined social groups. Both are important and, in this sense, the authors propose that “the universal is not the starting point, but the end point” (Rosenfield and Pauli, 2012, p. 322), that is, both contextual elements and universal elements are extremely important for decent work.

Hence, the research participants from the Brazilian context attempt to work for fair income, social protection, security and better personal development opportunities, as the ILO recommends (International Labour Office [ILO], 1999). These elements are juxtaposed with the universally established collective discourse the ILO puts forward and the specific social discourse of the Brazilian context, constructed based on the singular narratives of the participant group, which can represent potential universal elements of the decent work discourse.

However, the main difference between the ILO’s discourse and the participants’ narratives indicates that the elements described earlier as potentially universal do not come from the action of the State, like in the formal employment and qualification model, but from the informal economy, composed of mainly family and community relationship networks. Hence, the context-specific concepts inherent to decent work, in a more concrete form of the narratives of the participants from the city of São Paulo, Brazil, is that working opportunities, as well as social protection, are produced by informal relationship networks; qualification takes place through informal learning in practice from more experienced colleagues; success and security are perceived as the opportunity to continue working (employed or working in informality); and personal and professional development is defined as the possibility to make choices and have control over one’s life.

These findings show the need to focus on understanding the daily reality of “unseen” people (Spink, 2007), granting them voice in our studies (Blustein, 2006) in an attempt to define what decent work means to these persons, not based on an idealized view of paid and stable working, but based on what emerges concretely.

## Conclusion

In response to the questions formulated in the introduction to this article, the participants in this study coming from the Brazilian reality of the city of São Paulo produced four conclusions. First, the notion of dignity associated with decent work should be a synthesis of the tension between the universal and the particular. Second, the decent work model should not be based exclusively on the model of typical working, out of respect for contexts in which that is not the predominant working model and alternative working forms have been produced, such as in the case of the Brazilian context, particularly the city of São Paulo. Third, the concept of decent work that should be adopted, should contain universal elements, but these elements should be relativized as a function of the context in which working happens from a psychosocial perspective of decent work (plural concepts of decent work). And, fourth, in contexts in which informality structures the working world, decent work should be advanced in the formal as well as informal economy.

In conclusion, the synthesis of the participants’ singular narratives indicated the presence of the principles the International Labour Office [ILO] (1999) recommended. However, in more collective contexts with vulnerability and restricted State support, these principles are co-constructed in the community, but not offered by the public power, which produces a distinguished form of decent work.

The main contribution this study offers is the analysis of the concept of decent work through the narratives of workers who do not make a clear distinction between formal and informal working, and who works in collective contexts marked by inequality, vulnerability, and partial absence of the State as a source of security and social protection.

However, the core limitations lie in the analysis occurring in a more collective and unequal context, which somewhat diminishes the relevance of the findings for individual and equalitarian contexts with strong State action to guarantee decent work. Further, gender, race and social class were not analyzed, which intersect with and constitute the experiences of the study participants. That said, the issue of gender inequality in the working world clearly emerged in the narratives of the women interviewed and issues of social class and race determined the career construction possibilities of the participants. Moreover, the discussion about work stress, burnout and work addiction, in addition to an analysis about work health, work conditions, and the contribution of these concepts in the definition of decent work was not presented.

Quantitative studies and similar research with other sets of workers, mainly analyzing intersectionality between gender, race, and social class would be very important.

## Author Contributions

All authors listed, have made substantial, direct and intellectual contribution to the work, and approved it for publication.

## Conflict of Interest Statement

The authors declare that the research was conducted in the absence of any commercial or financial relationships that could be construed as a potential conflict of interest.

## Footnotes

1 In the Brazilian context, the term “mulatto” is used to designate everyone who is son of white man and black woman or vice versa.
